# *catena*-Poly[[[bis­(quinoxaline-2-carboxyl­ato-κ^2^*N*^1^,*O*)zinc(II)]-μ_2_-1,2-bis­(pyridin-4-yl)ethene-κ^2^*N*:*N*′] hemihydrate]

**DOI:** 10.1107/S2414314625007527

**Published:** 2025-09-05

**Authors:** Rund I. Shehadeh, Mason F. Hester, Oliver A. Cernero, Henry C. Neal, Bradley W. Smucker

**Affiliations:** a 900 N Grand Avenue, Suite 61651, Sherman, TX 75090, USA; Benemérita Universidad Autónoma de Puebla, México

**Keywords:** crystal structure, zinc(II), polymeric chains, quinoxaline

## Abstract

The title complex, {[Zn(C_9_H_5_N_2_O_2_)_2_(C_12_H_10_N_2_)]·0.5H_2_O}_*n*_, has six-coordinate Zn^II^ ions with two *trans*-bidentate quinoxaline-2-carboxyl­ato ligands and a bridging 1,2-bis­(pyridin-4-yl)ethyl­ene ligand, resulting in polymeric chains along the [101] direction. The offset packing of these one-dimensional chains gives rise to inter-chain H⋯O and H⋯ring inter­actions.

## Abstract 2

In the reported structure, Zn^II^ centres with two *trans*-quinoxaline-2-carboxyl­ato ligands are bridged by 1,2-bis­(pyridin-4-yl)ethyl­ene to form polymeric chains.

## Graphical abstract

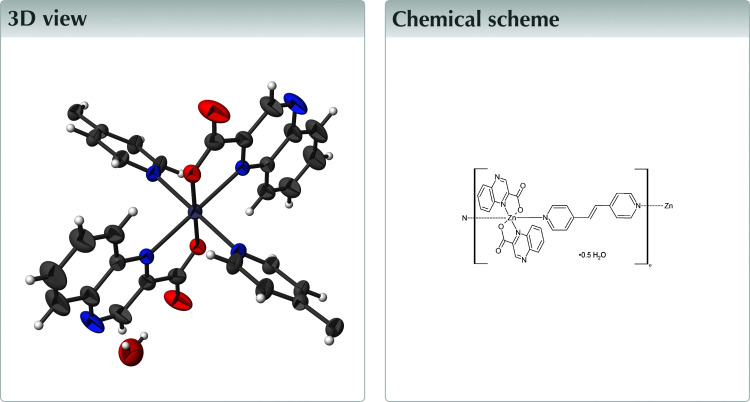

## Structure description

**Figure 1:**
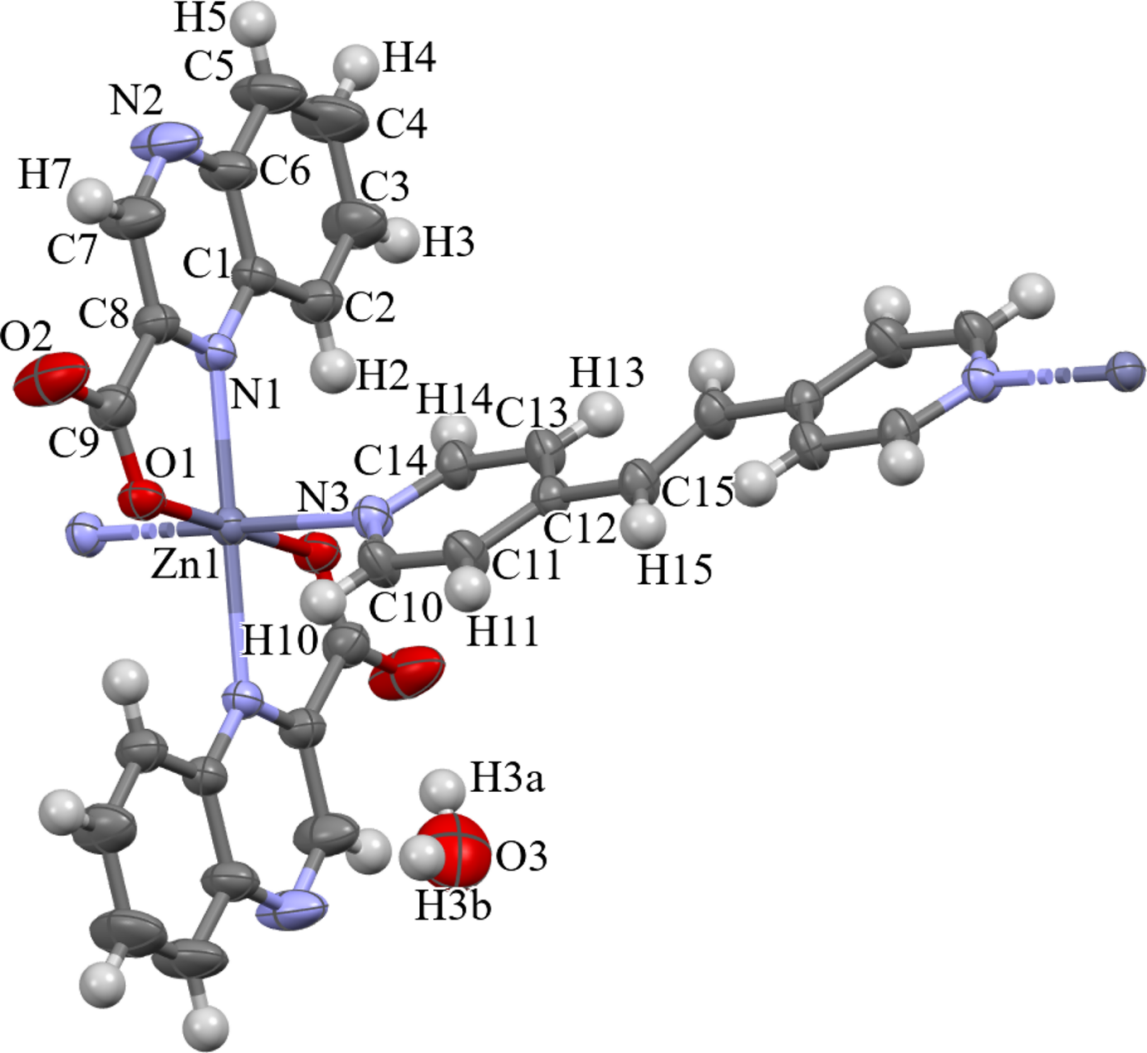
Displacement ellipsoid plot (50% probability) of a single repeating unit of the title compound with the distance between atom O2 and the H3*a* of the inter­stitial water mol­ecule shown.

**Figure 2:**
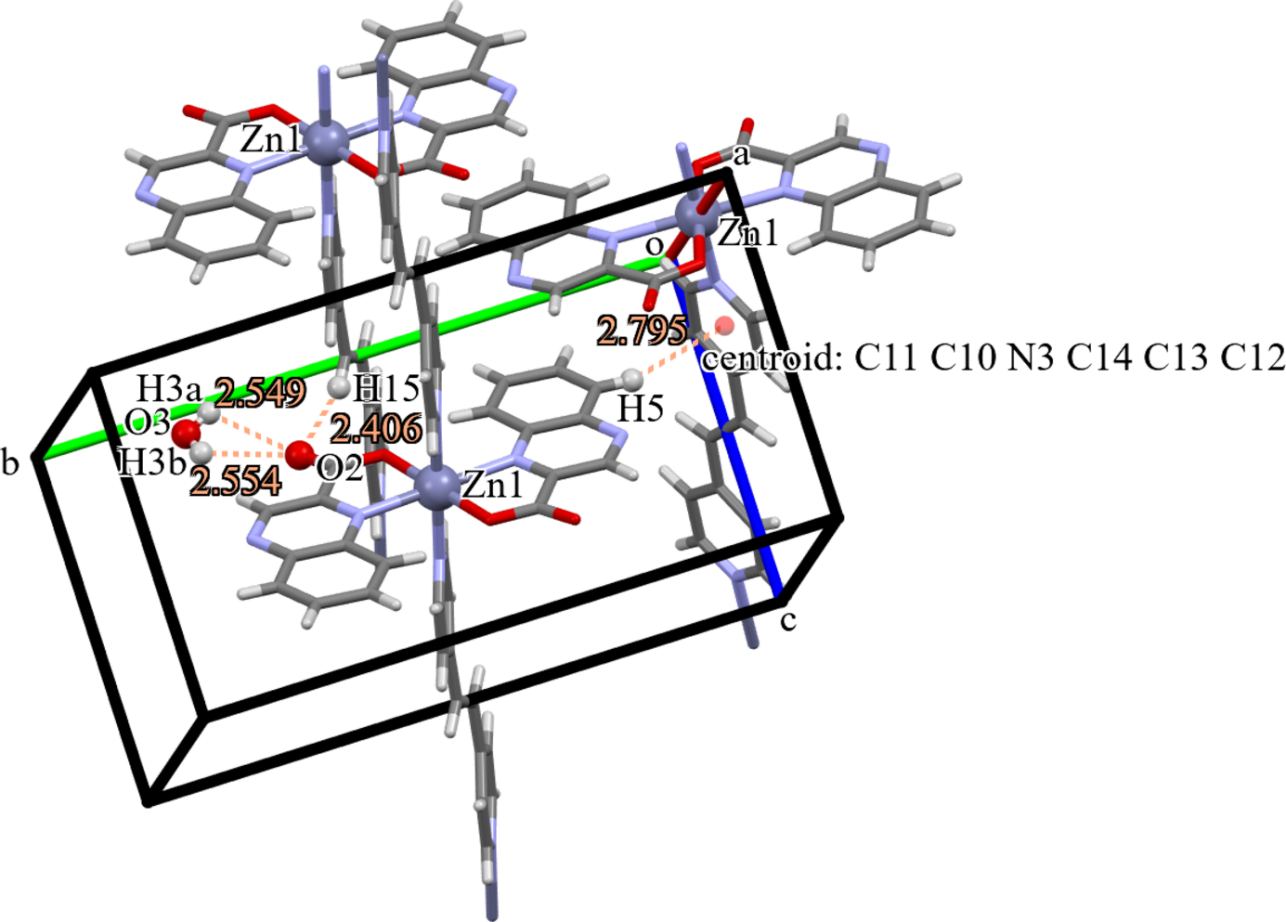
Partial packing of the title complex using capped or ball-and-stick models with the inter­molecular H⋯O distances between O2 and atoms H3*a*, H3*b* and H15(*x*, *y*, *z* − 1), as well as the H⋯centroid distance between H5(−*x* + 1, −*y* + 1, −*z* + 1) and the centroid of the pyridyl ring N3/C10⋯C14(*x*, −*y* + 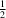, *z* − 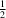).

The single repeating unit of the polymeric title complex has a six-coordinated zinc(II) ion lying on an inversion centre, with two *trans*-bidentate quinoxaline-2-carboxyl­ate ligands and a 1,2-bis­(pyridin-4-yl)ethyl­ene ligand bridging each zinc centre (Fig. 1 ▸). The resulting polymeric chains propagate along the [10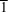] direction, with each one-dimensional chain offset from another by a half of the 1,2-bis­(pyridin-4-yl)ethyl­ene (Fig. 2 ▸). The Zn⋯Zn distances within each polymer is 13.7278 (5) Å and between offset polymers is 9.1330 (3) Å, which corresponds to the *c* dimension of the unit cell. This staggered packing gives rise to inter-chain close proximity of H15(*x*, *y*, *z* − 1) and O2, 2.406 (3) Å. Additionally, the H5(1 − *x*, 1 − *y*, 1 − *z*) on the quinoxaline is positioned near the π system of the pyridyl ring with the H5⋯centroid[(N3/C10⋯C14)(*x*, 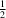 − *y*, −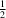 + *z*)] distance of 2.795 Å (Fig. 2 ▸), which is similar to the range of H⋯centroid distances, 2.73–3.03 Å, observed in the sandwich packing of water between two C_6_ rings (Dong *et al.*, 2016 ▸). The H atoms of the lattice water mol­ecule are situated so as to hydrogen bond with O2 (Table 1 ▸, Fig. 2 ▸).

This *N*,*O*-bidentate quinoxaline-2-carboxyl­atate ligand, qlc, has also been employed with copper(II) to form mol­ecular [Cu(qlc)_2_(H_2_O)_2_] (Feng *et al.*, 2007 ▸). Another derivative of the qlc ligand is 3-hy­droxy-2-quinoxaline­carboxyl­ate, hqxc. As an equatorial bidentate ligand with zinc(II), hqxc has been used to generate mol­ecular complexes with *trans* pyridine or DMSO ligands (Sakai *et al.*, 2010 ▸), or to form a polymeric complex using bridging *trans* 4,4-bi­pyridine (Xiao *et al.*, 2013 ▸), which forms similar offset polymer chains as the title compound.

## Synthesis and crystallization

A DMF solution of 7 mg of ZnCl_2_ and 17 mg of 2-quinoxaline­carb­oxy­lic acid was heated at 393 K for 1 d. After cooling, 7 mg of 1,10-methyl­enebis{4-[(*E*)-2-(pyridin-4-yl)vin­yl]pyridinium} bis­(hexa­fluoro­phosphate) (Blanco *et al.*, 2009 ▸) was added and the solution heated for a few days, then left to air cool in an oven for seven months. Dichroic brown/blue crystals of the title compound were harvested from the solution. Thermal decomposition of the pyridinium salt to *trans*-1,2-bis­(pyridin-4-yl)ethyl­ene likely occurred. Water of crystallization likely originated from prolonged air exposure.

## Refinement

Crystal data, data collection, and structure refinement details are summarized in Table 2 ▸. The lattice water mol­ecule was refined with a site occupancy factor fixed to 1/4, in such a way that the monomeric formula for the Zn^II^ complex is hemi-hydrated, since the Zn^II^ ion is placed on an inversion centre.

## Supplementary Material

Crystal structure: contains datablock(s) I. DOI: 10.1107/S2414314625007527/bh4099sup1.cif

Structure factors: contains datablock(s) I. DOI: 10.1107/S2414314625007527/bh4099Isup2.hkl

CCDC reference: 2481934

Additional supporting information:  crystallographic information; 3D view; checkCIF report

## Figures and Tables

**Table 1:** Hydrogen-bond geometry (Å, °)

*D*—H⋯*A*	*D*—H	H⋯*A*	*D*⋯*A*	*D*—H⋯*A*
C2—H2⋯O1^i^	0.93 (1)	2.41 (1)	3.250 (3)	151 (1)
C15—H15⋯O2^ii^	0.93 (1)	2.41 (1)	3.307 (3)	163 (1)

Symmetry codes: (i) 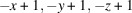; (ii) 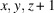.

**Table 2:** Experimental details

Crystal data	
Chemical formula	[Zn(C_9_H_5_N_2_O_2_)_2_(C_12_H_10_N_2_)]·0.5H_2_O
*M*_r_	602.90
Crystal system, space group	Monoclinic, *P*2_1_/*c*
Temperature (K)	293
*a*, *b*, *c* (Å)	8.9506 (3), 17.1862 (6), 9.1330 (3)
β (°)	98.770 (3)
*V* (Å^3^)	1388.47 (8)
*Z*	2
Radiation type	Mo *K*α
μ (mm^−1^)	0.93
Crystal size (mm)	0.51 × 0.28 × 0.26

Data collection	
Diffractometer	XtaLAB Mini II
Absorption correction	Analytical (*CrysAlis PRO*; Rigaku OD, 2024 ▸)
*T*_min_, *T*_max_	0.626, 0.758
No. of measured, independent and observed [*I* > 2σ(*I*)] reflections	20662, 4146, 2813
*R*_int_	0.039
(sin θ/λ)_max_ (Å^−1^)	0.716

Refinement	
*R*[*F*^2^ > 2σ(*F*^2^)], *wR*(*F*^2^), *S*	0.042, 0.099, 1.00
No. of reflections	4146
No. of parameters	199
H-atom treatment	H-atom parameters constrained
Δρ_max_, Δρ_min_ (e Å^−3^)	0.44, −0.44

Computer programs: *CrysAlis PRO* (Rigaku OD, 2024 ▸), *SHELXT* (Sheldrick, 2015 ▸), *OLEX2.refine* (Bourhis *et al.*, 2015 ▸), *Mercury* (Macrae *et al.*, 2020 ▸), *OLEX2* (Dolomanov *et al.*, 2009 ▸) and *publCIF* (Westrip, 2010 ▸).

## full crystallographic data

### catena-Poly[[[bis(quinoxaline-2-carboxylato-κ2N1,O)zinc(II)]-µ2-1,2-bis(pyridin-4-yl)ethene-κ2N:N'] hemihydrate] Crystal data

**Table:**

[Zn(C_9_H_5_N_2_O_2_)_2_(C_12_H_10_N_2_)]·0.5H_2_O	*F*(000) = 619.085
*M**_r_* = 602.90	*D*_x_ = 1.442 Mg m^−^^3^
Monoclinic, *P*2_1_/*c*	Mo *K*α radiation, λ = 0.71073 Å
*a* = 8.9506 (3) Å	Cell parameters from 5554 reflections
*b* = 17.1862 (6) Å	θ = 2.3–24.6°
*c* = 9.1330 (3) Å	µ = 0.93 mm^−^^1^
β = 98.770 (3)°	*T* = 293 K
*V* = 1388.47 (8) Å^3^	Prism, light yellow
*Z* = 2	0.51 × 0.28 × 0.26 mm

### catena-Poly[[[bis(quinoxaline-2-carboxylato-κ2N1,O)zinc(II)]-µ2-1,2-bis(pyridin-4-yl)ethene-κ2N:N'] hemihydrate] Data collection

**Table:**

XtaLAB Mini II diffractometer	4146 independent reflections
Radiation source: fine-focus sealed X-ray tube, Rigaku (Mo) X-ray Source	2813 reflections with *I* > 2σ(*I*)
Graphite monochromator	*R*_int_ = 0.039
Detector resolution: 10.0000 pixels mm^-1^	θ_max_ = 30.6°, θ_min_ = 2.4°
ω scans	*h* = −12→12
Absorption correction: analytical (CrysAlis PRO; Rigaku OD, 2024)	*k* = −24→24
*T*_min_ = 0.626, *T*_max_ = 0.758	*l* = −12→12
20662 measured reflections	

### catena-Poly[[[bis(quinoxaline-2-carboxylato-κ2N1,O)zinc(II)]-µ2-1,2-bis(pyridin-4-yl)ethene-κ2N:N'] hemihydrate] Refinement

**Table:**

Refinement on *F*^2^	23 constraints
Least-squares matrix: full	Primary atom site location: dual
*R*[*F*^2^ > 2σ(*F*^2^)] = 0.042	H-atom parameters constrained
*wR*(*F*^2^) = 0.099	*w* = 1/[σ^2^(*F*_o_^2^) + (0.0401*P*)^2^ + 0.5115*P*] where *P* = (*F*_o_^2^ + 2*F*_c_^2^)/3
*S* = 1.00	(Δ/σ)_max_ = 0.0004
4146 reflections	Δρ_max_ = 0.44 e Å^−^^3^
199 parameters	Δρ_min_ = −0.44 e Å^−^^3^
0 restraints	

### catena-Poly[[[bis(quinoxaline-2-carboxylato-κ2N1,O)zinc(II)]-µ2-1,2-bis(pyridin-4-yl)ethene-κ2N:N'] hemihydrate] Fractional atomic coordinates and isotropic or equivalent isotropic displacement parameters (Å^2^)

**Table:**

	*x*	*y*	*z*	*U*_iso_*/*U*_eq_	Occ. (<1)
Zn1	0.5	0.5	0.5	0.02940 (10)	
O1	0.33184 (15)	0.54080 (8)	0.34057 (15)	0.0357 (3)	
N1	0.54028 (17)	0.62863 (9)	0.51597 (17)	0.0309 (4)	
N3	0.34623 (18)	0.51175 (9)	0.66291 (17)	0.0323 (4)	
C12	0.1566 (2)	0.51820 (11)	0.8812 (2)	0.0306 (4)	
C13	0.1040 (2)	0.49021 (11)	0.7382 (2)	0.0334 (4)	
H13	0.0049 (2)	0.47303 (11)	0.7134 (2)	0.0401 (5)*	
O2	0.2120 (2)	0.64790 (10)	0.2512 (2)	0.0799 (7)	
C11	0.3046 (2)	0.54536 (13)	0.9078 (2)	0.0401 (5)	
H11	0.3429 (2)	0.56663 (13)	0.9995 (2)	0.0481 (6)*	
C15	0.0653 (2)	0.51873 (12)	1.0016 (2)	0.0350 (5)	
H15	0.1013 (2)	0.54724 (12)	1.0862 (2)	0.0420 (5)*	
C1	0.6463 (2)	0.67477 (11)	0.6011 (2)	0.0353 (4)	
C14	0.2015 (2)	0.48856 (11)	0.6352 (2)	0.0341 (4)	
H14	0.1648 (2)	0.47029 (11)	0.5407 (2)	0.0410 (5)*	
C8	0.4346 (2)	0.66382 (12)	0.4235 (2)	0.0376 (5)	
C9	0.3153 (2)	0.61372 (13)	0.3298 (2)	0.0409 (5)	
C10	0.3948 (2)	0.54077 (13)	0.7980 (2)	0.0402 (5)	
H10	0.4939 (2)	0.55865 (13)	0.8188 (2)	0.0483 (6)*	
C2	0.7619 (3)	0.64133 (13)	0.7037 (3)	0.0444 (5)	
H2	0.7673 (3)	0.58763 (13)	0.7158 (3)	0.0533 (6)*	
N2	0.5273 (3)	0.79219 (12)	0.4877 (3)	0.0666 (7)	
C3	0.8666 (3)	0.68857 (15)	0.7855 (3)	0.0637 (8)	
H3	0.9435 (3)	0.66659 (15)	0.8527 (3)	0.0765 (9)*	
C6	0.6378 (3)	0.75669 (13)	0.5851 (3)	0.0492 (6)	
C7	0.4294 (3)	0.74598 (13)	0.4103 (3)	0.0586 (7)	
H7	0.3525 (3)	0.76813 (13)	0.3432 (3)	0.0703 (8)*	
C5	0.7479 (3)	0.80324 (15)	0.6718 (3)	0.0733 (9)	
H5	0.7441 (3)	0.85710 (15)	0.6621 (3)	0.0879 (10)*	
C4	0.8589 (3)	0.76983 (16)	0.7688 (4)	0.0799 (10)	
H4	0.9308 (3)	0.80101 (16)	0.8251 (4)	0.0959 (12)*	
O3	0.1505 (13)	0.7936 (6)	0.0902 (12)	0.102 (3)	0.250000
H3a	0.133 (19)	0.749 (4)	0.050 (16)	0.153 (5)*	0.250000
H3b	0.220 (15)	0.789 (8)	0.165 (13)	0.153 (5)*	0.250000

### catena-Poly[[[bis(quinoxaline-2-carboxylato-κ2N1,O)zinc(II)]-µ2-1,2-bis(pyridin-4-yl)ethene-κ2N:N'] hemihydrate] Atomic displacement parameters (Å^2^)

**Table:**

	*U*^11^	*U*^22^	*U*^33^	*U*^12^	*U*^13^	*U*^23^
Zn1	0.03042 (16)	0.02834 (16)	0.03095 (16)	−0.00198 (14)	0.00956 (11)	−0.00164 (14)
O1	0.0373 (8)	0.0316 (7)	0.0370 (7)	−0.0035 (6)	0.0021 (6)	−0.0036 (6)
N1	0.0315 (8)	0.0273 (8)	0.0349 (9)	−0.0034 (6)	0.0083 (7)	−0.0033 (7)
N3	0.0323 (8)	0.0356 (10)	0.0309 (8)	−0.0017 (7)	0.0106 (6)	−0.0014 (7)
C12	0.0315 (10)	0.0310 (10)	0.0308 (9)	0.0002 (7)	0.0100 (7)	−0.0001 (7)
C13	0.0262 (9)	0.0429 (12)	0.0317 (9)	−0.0037 (8)	0.0063 (7)	−0.0007 (8)
O2	0.0717 (13)	0.0456 (11)	0.1053 (16)	0.0108 (9)	−0.0416 (12)	−0.0095 (10)
C11	0.0398 (11)	0.0504 (13)	0.0321 (10)	−0.0111 (10)	0.0116 (9)	−0.0111 (9)
C15	0.0346 (10)	0.0440 (12)	0.0281 (9)	−0.0023 (8)	0.0101 (8)	−0.0050 (8)
C1	0.0343 (11)	0.0293 (10)	0.0429 (11)	−0.0041 (8)	0.0077 (9)	−0.0041 (9)
C14	0.0327 (10)	0.0431 (12)	0.0274 (9)	−0.0005 (8)	0.0070 (7)	−0.0022 (8)
C8	0.0381 (11)	0.0295 (10)	0.0447 (12)	−0.0009 (9)	0.0047 (9)	−0.0034 (9)
C9	0.0379 (11)	0.0380 (12)	0.0453 (12)	0.0024 (9)	0.0011 (9)	−0.0045 (10)
C10	0.0329 (11)	0.0492 (13)	0.0409 (11)	−0.0120 (9)	0.0132 (9)	−0.0108 (10)
C2	0.0437 (12)	0.0336 (11)	0.0540 (13)	−0.0013 (9)	0.0012 (10)	−0.0045 (10)
N2	0.0731 (16)	0.0277 (10)	0.0897 (17)	−0.0030 (10)	−0.0167 (13)	0.0007 (10)
C3	0.0544 (16)	0.0477 (15)	0.0794 (19)	−0.0050 (12)	−0.0208 (14)	−0.0080 (13)
C6	0.0514 (14)	0.0305 (11)	0.0625 (16)	−0.0078 (10)	−0.0014 (12)	−0.0040 (11)
C7	0.0626 (17)	0.0319 (12)	0.0735 (18)	0.0016 (11)	−0.0144 (13)	0.0031 (12)
C5	0.0778 (19)	0.0340 (13)	0.099 (2)	−0.0169 (13)	−0.0166 (17)	−0.0072 (14)
C4	0.071 (2)	0.0485 (16)	0.107 (2)	−0.0198 (14)	−0.0284 (18)	−0.0109 (16)
O3	0.108 (9)	0.108 (8)	0.094 (8)	0.005 (6)	0.026 (6)	0.035 (6)

### catena-Poly[[[bis(quinoxaline-2-carboxylato-κ2N1,O)zinc(II)]-µ2-1,2-bis(pyridin-4-yl)ethene-κ2N:N'] hemihydrate] Geometric parameters (Å, º)

**Table:**

Zn1—O1	2.0515 (13)	C15—H15	0.9300
Zn1—O1^i^	2.0515 (13)	C1—C2	1.408 (3)
Zn1—N1^i^	2.2411 (16)	C1—C6	1.416 (3)
Zn1—N1	2.2411 (16)	C14—H14	0.9300
Zn1—N3	2.1848 (15)	C8—C9	1.528 (3)
Zn1—N3^i^	2.1848 (15)	C8—C7	1.417 (3)
O1—C9	1.264 (2)	C10—H10	0.9300
N1—C1	1.382 (2)	C2—H2	0.9300
N1—C8	1.315 (3)	C2—C3	1.372 (3)
N3—C14	1.342 (2)	N2—C6	1.368 (3)
N3—C10	1.340 (2)	N2—C7	1.308 (3)
C12—C13	1.404 (3)	C3—H3	0.9300
C12—C11	1.390 (3)	C3—C4	1.405 (4)
C12—C15	1.467 (3)	C6—C5	1.414 (3)
C13—H13	0.9300	C7—H7	0.9300
C13—C14	1.378 (3)	C5—H5	0.9300
O2—C9	1.230 (3)	C5—C4	1.354 (4)
C11—H11	0.9300	C4—H4	0.9300
C11—C10	1.382 (3)	O3—H3a	0.8500
C15—C15^ii^	1.331 (4)	O3—H3b	0.8501
			
O1^i^—Zn1—O1	180.0	C6—C1—N1	119.38 (19)
N1—Zn1—O1	78.60 (6)	C6—C1—C2	119.80 (19)
N1—Zn1—O1^i^	101.40 (6)	C13—C14—N3	123.96 (17)
N1^i^—Zn1—O1	101.40 (6)	H14—C14—N3	118.02 (10)
N1^i^—Zn1—O1^i^	78.60 (6)	H14—C14—C13	118.02 (11)
N1^i^—Zn1—N1	180.0	C9—C8—N1	118.25 (17)
N3^i^—Zn1—O1	91.06 (6)	C7—C8—N1	121.59 (19)
N3^i^—Zn1—O1^i^	88.94 (6)	C7—C8—C9	120.16 (19)
N3—Zn1—O1	88.94 (6)	O2—C9—O1	126.0 (2)
N3—Zn1—O1^i^	91.06 (6)	C8—C9—O1	116.82 (18)
N3—Zn1—N1	88.64 (5)	C8—C9—O2	117.2 (2)
N3—Zn1—N1^i^	91.36 (5)	C11—C10—N3	123.03 (19)
N3^i^—Zn1—N1	91.36 (5)	H10—C10—N3	118.48 (11)
N3^i^—Zn1—N1^i^	88.64 (5)	H10—C10—C11	118.48 (12)
N3^i^—Zn1—N3	180.0	H2—C2—C1	120.25 (12)
C9—O1—Zn1	117.32 (13)	C3—C2—C1	119.5 (2)
C1—N1—Zn1	133.84 (13)	C3—C2—H2	120.25 (15)
C8—N1—Zn1	108.60 (12)	C7—N2—C6	116.0 (2)
C8—N1—C1	117.55 (17)	H3—C3—C2	119.63 (15)
C14—N3—Zn1	122.29 (12)	C4—C3—C2	120.7 (2)
C10—N3—Zn1	120.76 (13)	C4—C3—H3	119.63 (15)
C10—N3—C14	116.93 (16)	N2—C6—C1	122.2 (2)
C11—C12—C13	116.91 (17)	C5—C6—C1	118.8 (2)
C15—C12—C13	123.61 (17)	C5—C6—N2	119.0 (2)
C15—C12—C11	119.48 (17)	N2—C7—C8	123.2 (2)
H13—C13—C12	120.49 (11)	H7—C7—C8	118.38 (13)
C14—C13—C12	119.01 (17)	H7—C7—N2	118.38 (14)
C14—C13—H13	120.49 (11)	H5—C5—C6	119.81 (15)
H11—C11—C12	119.97 (11)	C4—C5—C6	120.4 (2)
C10—C11—C12	120.07 (18)	C4—C5—H5	119.81 (15)
C10—C11—H11	119.97 (12)	C5—C4—C3	120.8 (2)
C15^ii^—C15—C12	124.6 (2)	H4—C4—C3	119.62 (15)
H15—C15—C12	117.69 (11)	H4—C4—C5	119.62 (15)
C2—C1—N1	120.82 (18)	H3b—O3—H3a	109.5
			
Zn1—O1—C9—O2	−172.45 (18)	C11—C12—C13—C14	2.2 (2)
Zn1—O1—C9—C8	7.37 (14)	C11—C12—C15—C15^ii^	−166.6 (2)
Zn1—N1—C1—C2	0.96 (19)	C11—C10—N3—C14	1.9 (3)
Zn1—N1—C1—C6	−178.84 (19)	C15—C12—C13—C14	−176.65 (19)
Zn1—N1—C8—C9	−0.49 (14)	C15—C12—C11—C10	176.0 (2)
Zn1—N1—C8—C7	179.21 (16)	C1—N1—C8—C9	−179.27 (18)
Zn1—N3—C14—C13	175.49 (14)	C1—N1—C8—C7	0.4 (2)
Zn1—N3—C10—C11	−176.25 (16)	C1—C2—C3—C4	0.5 (3)
O1—C9—C8—N1	−4.4 (2)	C1—C6—N2—C7	0.1 (3)
O1—C9—C8—C7	175.9 (2)	C1—C6—C5—C4	−0.3 (3)
N1—C1—C2—C3	179.3 (2)	C8—N1—C1—C2	179.35 (19)
N1—C1—C6—N2	0.2 (2)	C8—N1—C1—C6	−0.5 (2)
N1—C1—C6—C5	−179.3 (2)	C8—C7—N2—C6	−0.1 (3)
N1—C8—C9—O2	175.4 (2)	C9—C8—C7—N2	179.6 (2)
N1—C8—C7—N2	−0.1 (3)	C2—C1—C6—N2	−179.6 (2)
N3—C14—C13—C12	0.5 (2)	C2—C1—C6—C5	0.8 (3)
N3—C10—C11—C12	0.9 (3)	C2—C3—C4—C5	0.1 (4)
C12—C15—C15^ii^—C12^ii^	180.0 (3)	N2—C6—C5—C4	−179.9 (3)
C13—C12—C11—C10	−2.9 (2)	C3—C2—C1—C6	−0.9 (3)
C13—C12—C15—C15^ii^	12.3 (2)	C3—C4—C5—C6	−0.1 (4)
C13—C14—N3—C10	−2.6 (2)	C7—N2—C6—C5	179.6 (3)
O2—C9—C8—C7	−4.3 (3)		

Symmetry codes: (i) −*x*+1, −*y*+1, −*z*+1; (ii) −*x*, −*y*+1, −*z*+2.

### catena-Poly[[[bis(quinoxaline-2-carboxylato-κ2N1,O)zinc(II)]-µ2-1,2-bis(pyridin-4-yl)ethene-κ2N:N'] hemihydrate] Hydrogen-bond geometry (Å, º)

**Table:**

*D*—H···*A*	*D*—H	H···*A*	*D*···*A*	*D*—H···*A*
C2—H2···O1^i^	0.93 (1)	2.40 (1)	3.250 (3)	151 (1)
C15—H15···O2^iii^	0.93 (1)	2.41 (1)	3.307 (3)	163 (1)

Symmetry codes: (i) −*x*+1, −*y*+1, −*z*+1; (iii) *x*, *y*, *z*+1.
